# Supplementary material for: Tuning the Thermogelation and Rheology of Poly(2-Oxazoline)/Poly(2-Oxazine)s Based Thermosensitive Hydrogels for 3D Bioprinting
Source: Gels. 2021 Jun 24;7(3):78. doi: 10.3390/gels7030078 (PMC8293086; doi:10.3390/gels7030078)
Supplement: Supplementary file 1 [file gels-07-00078-s001.zip › gels-1216938-supplementary.pdf]

Supporting information

Malik Salman Haider [1,*], Taufiq Ahmad [2], Mengshi Yang [1], Chen Hu [1], Lukas Hahn [1], Philipp Stahlhut [2], Jürgen Groll [2] and Robert Luxenhofer [1,3,*]

[1] Functional Polymer Materials, Chair for Advanced Materials Synthesis, Institute for Functional Materials and Biofabrication, Department of Chemistry and Pharmacy, Julius-Maximilians-University Würzburg, Röntgenring 11, 97070 Würzburg, Germany; mengshi.yang@uni-wuerzburg.de (M.Y.); chen.hu@uni-wuerzburg.de (C.H.); lukas.hahn@uni-wuerzburg.de (L.H.)

[2] Department of Functional Materials in Medicine and Dentistry, Institute for Functional Materials and Biofabrication and Bavarian Polymer Institute, Julius-Maximilians-University Würzburg, Pleicherwall 2, 97070 Würzburg, Germany; taufiq.ahmad@fmz.uni-wuerzburg.de (T.A.); philipp.stahlhut@fmz.uni-wuerzburg.de (P.S.); juergen.groll@fmz.uni-wuerzburg.de (J.G.)

[3] Soft Matter Chemistry, Department of Chemistry and Helsinki Institute of Sustainability Science, Faculty of Science, University of Helsinki, PB 55, 00014 Helsinki, Finland

* Correspondence: malik.haider@uni-wuerzburg.de (M.S.H.); robert.luxenhofer@helsinki.fi (R.L.)

Table of Contents:

Synthesis of pPrOzi-*b*-pEtOx 50 repeat units

Synthesis of pPrOzi-*b*-pEtOx 100 repeat units

Figure S1. Heat flow occurring during the various heating and cooling cycle (10 K/min) of differential scanning calorimetry of pPrOzi$_{100}$-*b*-pMeOx$_{100}$, green vertical line indicating the glass transition point.

Figure S2. a) Amplitude and b) frequency sweep of pPrOzi$_{100}$-*b*-pEtOx$_{100}$ diblock copolymer at 20 and 15 wt.% concentration at constant temperature of 25°C.

Figure S3. SEM image of 30 wt.% pPrOzi$_{100}$-*b*-pEtOx$_{100}$ diblock copolymer lyophilized hydrogel (Top view).

Figure S4. a) 3D printability of 25 wt.% pPrOzi$_{100}$-*b*-pEtOx$_{100}$ based hydrogel, a 5 layered star (pressure; 90kPa, speed 2mm/sec). b) cryo-SEM image of the 25 wt.% pPrOzi$_{100}$-*b*-pEtOx$_{100}$ diblock copolymer with 1 wt.% laponite XLG.

Figure S5. Comparison of rheological properties of pristine (25 wt.%) and hybrid hydrogel (25 + 1 wt.% clay) prepared in water including a) temperature dependent sweep (from 5 to 60°C), b) amplitude sweep, c) yield stress and flow point, d) frequency sweep, e) shear thinning and f) structure recovery properties (at 25°C).

Figure S6. Visual appearance of 30 wt.% pPrOzi$_{100}$-*b*-pEtOx$_{100}$ diblock copolymer hydrogel in water (blue) and cell culture media (magenta). Comparison of rheological properties of hydrogel prepared in water and media including temperature dependent sweep (from 5 to 60°C), frequency sweep, angular sweep, shear thinning and structure recovery properties (at 25°C).

Figure S7. Live/dead viability assay of post-printing cell laden bioink a) serpentine line and b) a star showing the dead cells (red). Arrows indicating the dead cells and dashed lines are added for eye guidance.

Synthesis of pPrOzi$_{50}$-*b*-pEtOx$_{50}$ diblock copolymer

Initiation: MeOTf 0.0722 g (0.44 mmol; 1 eq)

1. Block: PrOzi 2.77 g (21.8 mmol; 50 eq)

2. Block: EtOx 2.16g (21.8 mmol; 50 eq)

Termination: EPC 0.22 g (1.42 mmol; 3.2 eq) (Ethyl isonipicotate)

Solvent Benzonitrile 9 ml

Yield: 4.5 g of white powder 84%

GPC (HFIP) M_n = 4.5 kg/mol; Đ = 1.3

^{1}H-NMR M_n = 11.3 kg/mol (pPrOzi$_{47}$- pEtOx$_{52}$)

Synthesis of pPrOzi$_{100}$-*b*-pEtOx$_{100}$ diblock copolymer

Initiation: MeOTf 0.0718 g (0.437 mmol; 1 eq)

1. Block: PrOzi 5.56 g (43.79 mmol; 100 eq)

2. Block: EtOx 4.34 g (43.79 mmol; 100 eq)

Termination: BocPip 0.245 g (1.31 mmol; 3 eq)

K_2CO_3 0.06 g (0.43 mmol; 1 eq)

Solvent Benzonitrile 10 ml

Yield: 7 g of white powder 70 %

GPC (HFIP) M_n = 9.1 kg/mol; Đ = 1.40

^{1}H-NMR M_n = 25.9 kg/mol (pPrOzi$_{112}$- pEtOx$_{117}$)

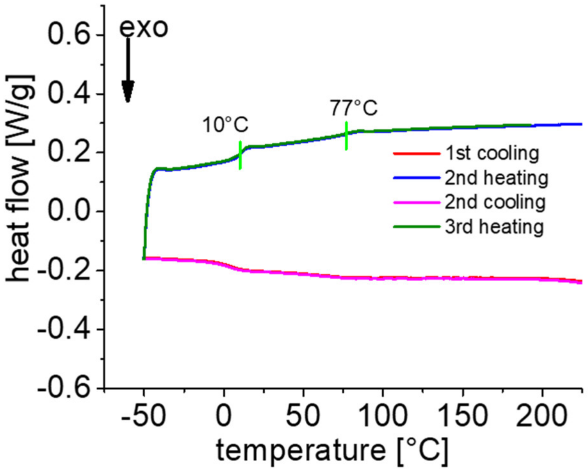

Figure S1. Heat flow occurring during the various heating and cooling cycle (10 K/min) of differential scanning calorimetry of pPrOzi100-*b*-pMeOx100, green vertical line indicating the glass transition point.

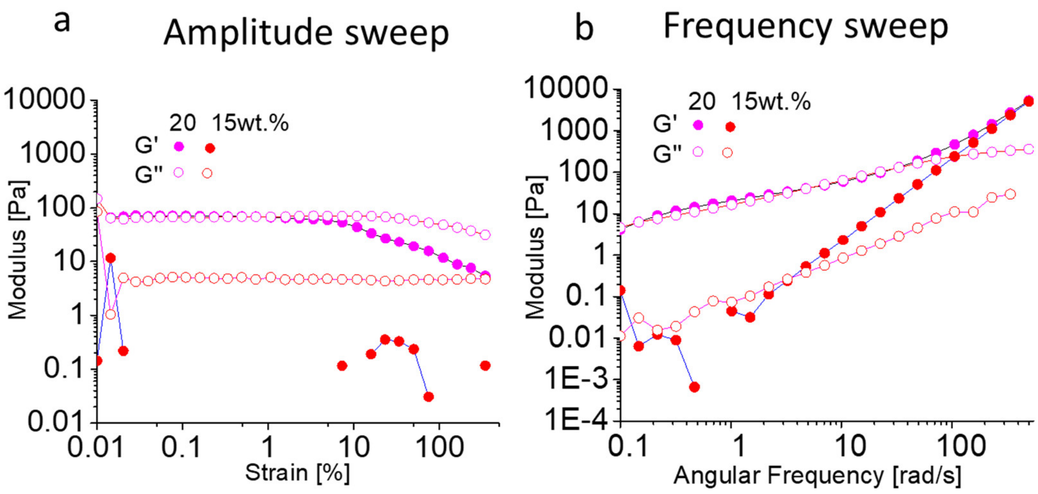

Figure S2. a) Amplitude and b) frequency sweep of pPrOzi100-*b*-pEtOx100 diblock copolymer at 20 and 15 wt.% concentration at constant temperature of 25°C.

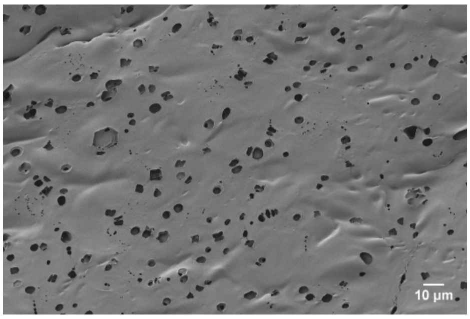

Figure S3. SEM image of 30 wt.% pPrOzi100-*b*-pEtOx100 diblock copolymer lyophilized hydrogel (Top view).

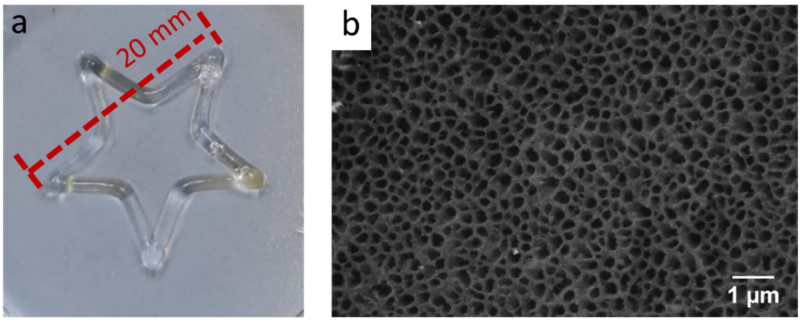

Figure S4. a) 3D printability of 25 wt.% pPrOzi100-*b*-pEtOx100 based hydrogel, a 5 layered star (pressure; 90kPa, speed 2mm/sec). b) cryo-SEM image of the 25 wt.% pPrOzi100-*b*-pEtOx100 diblock copolymer with 1 wt.% laponite XLG. .

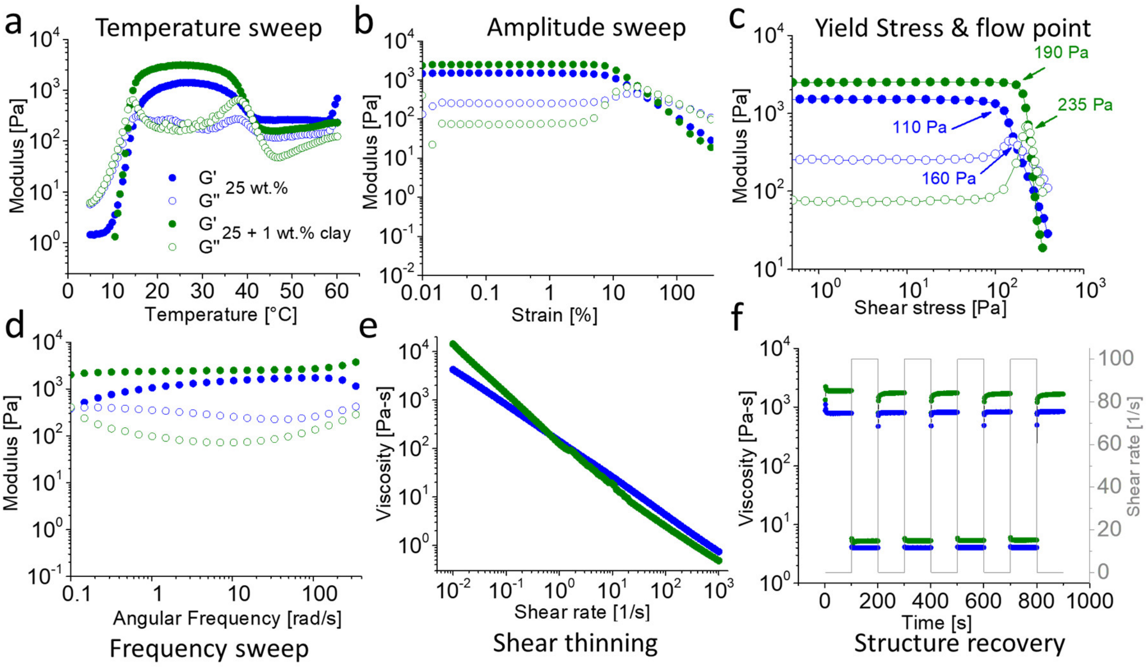

Figure S5. Comparison of rheological properties of pristine (25 wt.%) and hybrid hydrogel (25 + 1 wt.% clay) prepared in water including a) temperature dependent sweep (from 5 to 60°C), b) amplitude sweep, c) yield stress and flow point, d) frequency sweep, e) shear thinning and f) structure recovery properties (at 25°C).

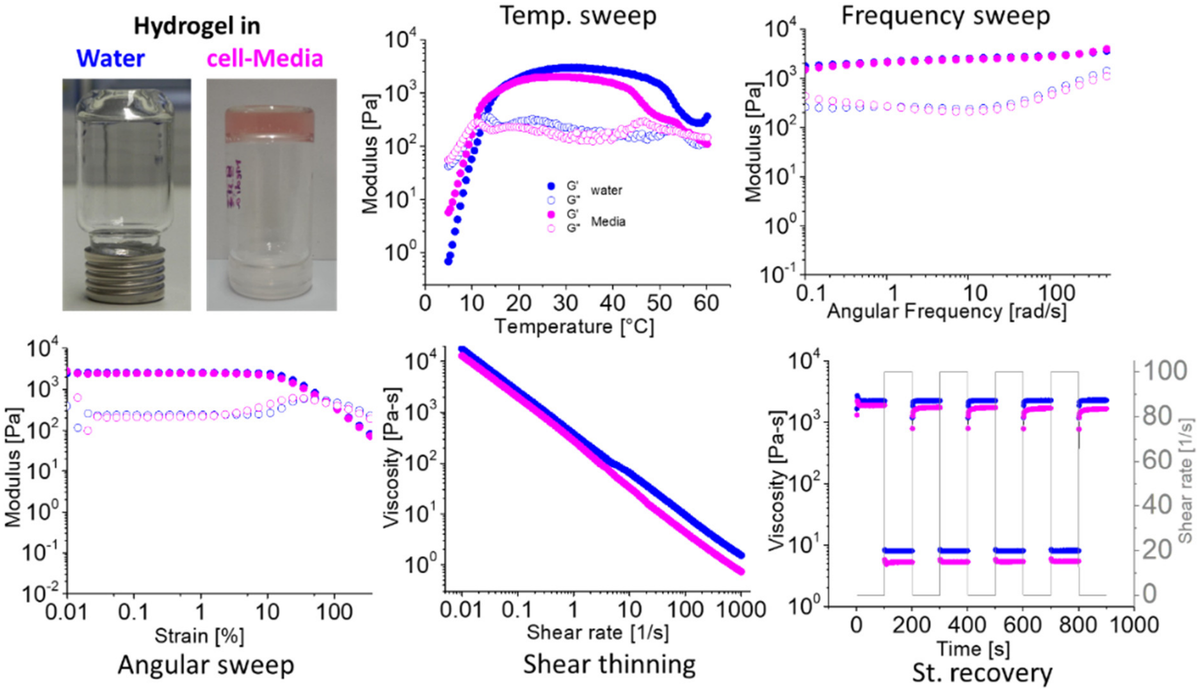

Figure S6. Visual appearance of 30 wt.% pPrOzi100-*b*-pEtOx100 diblock copolymer hydrogel in water (blue) and cell culture media (magenta). Comparison of rheological properties of hydrogel prepared in water and media including temperature dependent sweep (from 5 to 60°C), frequency sweep, angular sweep, shear thinning and structure recovery properties (at 25°C).

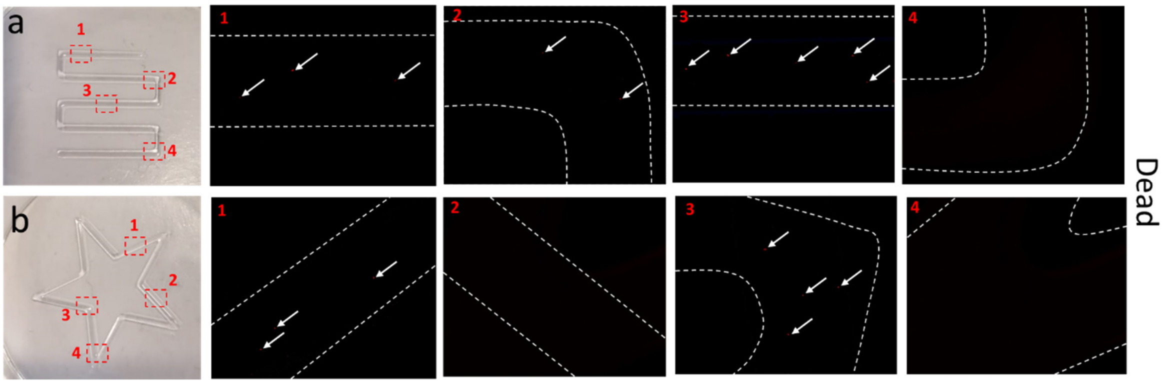

Figure S7. Live/dead viability assay of post-printing cell laden bioink a) serpentine line and b) a star showing the dead cells (red). Arrows indicating the dead cells and dashed lines are added for eye guidance.
